# Complete Genome Sequence of *Bacillus cereus* Group Phage TsarBomba

**DOI:** 10.1128/genomeA.01178-15

**Published:** 2015-10-15

**Authors:** Ivan Erill, Steven M. Caruso

**Affiliations:** Department of Biological Sciences, University of Maryland, Baltimore County, Baltimore, Maryland, USA

## Abstract

The *Bacillus cereus* group bacteriophage TsarBomba, a double-stranded DNA *Myoviridae*, was isolated from soil collected in Saratov, Russia. TsarBomba was found to be similar to *Bacillus* phages BCP78 and BCU4, and to have a wide host range among *Bacillus cereus* group species.

## GENOME ANNOUNCEMENT

The *Myoviridae* bacteriophage TsarBomba was isolated from soil samples collected from Saratov, Russia (GPS coordinates 51°36′12.0″ N, 45°58′21.0″ E) and cultivated on *Bacillus thuringiensis* subsp. *kurstaki* ATCC 33679 (Btk), a member of the *Bacillus cereus* group together with other closely related Gram-positive, endospore-forming, rod-shaped, aerobic, and facultative anaerobic bacteria including *B. cereus, B. anthracis, B. mycoides, B. pseudomycoides*, and *B. weihenstephanensis* (1). This double-stranded DNA bacteriophage has an icosahedral head 91 nm in diameter and a 202 nm long contractile tail, and produced 1-mm turbid plaques on Btk when grown overnight at 30°C on trypticase soy agar. Isolation and analysis of the phage was carried out by undergraduate student researchers as part of the SEA-PHAGES program described previously (2). Additional information about this and other *Bacillus* phages isolated by undergraduate researchers can be found at http://bacillus.phagesdb.org/.

TsarBomba was shotgun sequenced at the Pittsburgh Bacteriophage Institute to approximately 577-fold coverage by Illumina Sequencing. Genomic analysis determined TsarBomba to have a linear genome of 162,486 bp, with 40.1% G+C content, and 6,364-bp direct terminal repeats. TsarBomba contains 267 genes, 20 of which code for tRNA.

Comparison of TsarBomba with other phages belonging to the proposed C cluster by genomic synteny and phylogenetic methods suggests the inclusion of a fourth sub-cluster containing *B. cereus* group phages BCP78 (GenBank accession no. JN797797.1), TsarBomba, BCU4 (accession no. JN797798.1), Hobo, and IceQueen (A. B. Sauder, M. R. Quin, A. Brouillette, S. M. Caruso, S. Cresawn, L. Lewis, K. Loesser-Caser, M. Pate, C. Scott, S. Stockwell, and L. Temple, submitted for publication).

The ability of TsarBomba to infect other *Bacillus* species was tested, and TsarBomba was found to have a wide host range in the *B. cereus* group, being able to infect other strains of *B. thuringiensis* (PS52A1, DSM-350, and *Al Hakam*) and *B. cereus* (Gibson and FD45, though not 14579), and *B. anthracis Sterne*. The phage was not able to infect tested *B. pumilus, B. subtilis, B. megaterium, B. globegii*, or *B. simplex* strains.

Analysis of translational codon usage bias in the phage genes with the scnRCA index (3) trained on the Btk genome suggests that the 20 tRNA-coding genes detected in its genome are not used to alter the host tRNA pool, as it has been suggested for other phages (4).

### Nucleotide sequence accession number.

The complete genome sequence of the *Bacillus* phage TsarBomba is available in GenBank with the accession number KT224359.
