# Erratum for Erill and Caruso, Complete Genome Sequence of *Bacillus cereus* Group Phage TsarBomba

**DOI:** 10.1128/genomeA.01458-15

**Published:** 2015-11-19

**Authors:** Ivan Erill, Steven M. Caruso

**Affiliations:** Department of Biological Sciences, University of Maryland, Baltimore County, Baltimore, Maryland, USA

## ERRATUM

Volume 3, no. 5, e01178-15, 2015. Page 1: The byline should read as given above.

